# Poly (ADP-Ribose) Polymerase 1 Regulates Cajal–Retzius Cell Development and Neural Precursor Cell Adhesion

**DOI:** 10.3389/fcell.2021.693595

**Published:** 2021-10-11

**Authors:** Megan M. Nelson, J. Damon Hoff, Mya L. Zeese, Gabriel Corfas

**Affiliations:** ^1^Kresge Hearing Research Institute and Department of Otolaryngology – Head and Neck Surgery, University of Michigan, Ann Arbor, MI, United States; ^2^Neuroscience Graduate Program, University of Michigan, Ann Arbor, MI, United States; ^3^Single Molecule Analysis in Real-Time Center, Department of Biophysics, University of Michigan, Ann Arbor, MI, United States; ^4^Department of Molecular, Cellular, and Developmental Biology, University of Michigan, Ann Arbor, MI, United States

**Keywords:** PARP1, cortical development, Reelin, neuronal adhesion, N-cadherin

## Abstract

Poly (ADP-ribose) polymerase 1 (PARP1) is a ubiquitously expressed enzyme that regulates DNA damage repair, cell death, inflammation, and transcription. PARP1 functions by adding ADP-ribose polymers (PAR) to proteins including itself, using NAD^+^ as a donor. This post-translational modification known as PARylation results in changes in the activity of PARP1 and its substrate proteins and has been linked to the pathogenesis of various neurological diseases. PARP1 KO mice display schizophrenia-like behaviors, have impaired memory formation, and have defects in neuronal proliferation and survival, while mutations in genes that affect PARylation have been associated with intellectual disability, psychosis, neurodegeneration, and stroke in humans. Yet, the roles of PARP1 in brain development have not been extensively studied. We now find that loss of PARP1 leads to defects in brain development and increased neuronal density at birth. We further demonstrate that PARP1 loss increases the expression levels of genes associated with neuronal migration and adhesion in the E15.5 cerebral cortex, including *Reln*. This correlates with an increased number of Cajal–Retzius (CR) cells *in vivo* and in cultures of embryonic neural progenitor cells (NPCs) derived from the PARP1 KO cortex. Furthermore, PARP1 loss leads to increased NPC adhesion to N-cadherin, like that induced by experimental exposure to Reelin. Taken together, these results uncover a novel role for PARP1 in brain development, i.e., regulation of CR cells, neuronal density, and cell adhesion.

## Introduction

Poly (ADP-ribose) polymerase 1 (PARP1) is a ubiquitously expressed enzyme that plays roles in a variety of key biological processes, including DNA repair, inflammation, transcription, and programmed cell death (Kraus, 2008; Krishnakumar and Kraus, 2010; Jubin et al., 2017). PARP1 exerts its functions by protein PARylation, a post-translational modification consisting of the covalent attachment of ADP-ribose polymers (PAR) to itself and other proteins using NAD^+^ as a donor (Krishnakumar and Kraus, 2010). Extensive evidence implicates PARP1 in a number of nervous system diseases, including neurodegenerative disorders (Mao and Zhang, 2021), ischemic stroke (Endres et al., 1997; Chiarugi, 2005), glioma (Galia et al., 2012; Murnyák et al., 2017), epilepsy (Kim et al., 2014), traumatic brain injury (Stoica et al., 2014), and psychiatric disorders (Szebeni et al., 2016). Pharmacological inhibition of PARP1 *in vivo* causes defects in long-term memory (Goldberg et al., 2009), while complete loss of PARP1 causes impaired short-term memory formation (Hong et al., 2019). Additionally, adult PARP1 KO mice have a reduced brain weight, altered neuronal proliferation within the brain’s dentate gyrus (Plane et al., 2012) and subventricular zone (Hong et al., 2019), and display schizophrenia-like behaviors, including defects in pre-pulse inhibition, decreased social interaction, and increased anxiety-like behaviors (Hong et al., 2019). Accordingly, human studies have linked mutations in genes affecting PARylation to episodic psychosis, intellectual disability, peripheral neuropathy, ataxia, and increased risk of stroke (Najmabadi et al., 2011; Danhauser et al., 2018; Meng et al., 2018; Durmus et al., 2021).

Despite evidence that PARP1 dysregulation contributes to aberrant brain function and related disorders in humans and mice, very few studies have examined the roles of PARP1 in brain development. It has been reported that PARP1 loss in mice causes enlarged ventricles at E14.5, increases cortical cell death at E16.5 and E18.5, and impairs the proliferation of neural stem cells derived from embryonic telencephalon (Hong et al., 2019). This study also found that PARP1 influences neural stem cell differentiation by repressing a glial cell fate *in vitro* (Hong et al., 2019). While it is apparent that loss of PARP1 has detrimental effects on brain development and function, little is known about its role in regulation of neuronal migration and cortical patterning. Furthermore, no studies have yet assessed the effect of PARP1 loss on gene expression in the embryonic brain.

Here, we show that PARP1 loss results in decreased cortical thickness and increased neuronal density at birth. These changes are associated with an increase in Cajal–Retzius (CR) cell abundance and *Reln* mRNA levels in the PARP1 KO embryonic brain and in neural progenitor cells (NPCs) derived from the embryonic mutant telencephalon. Accordingly, PARP1 KO NPCs show excess adhesion to N-cadherin, likely through Reelin signaling. Additionally, RNA-sequencing of the PARP1 KO E15.5 cortex demonstrates that PARP1 loss results in increased expression levels of many genes associated with neuronal migration and adhesion during embryonic brain development. Taken together, our findings uncover a new role for PARP1 in regulating CR cell development, neuronal density, and cell adhesion.

## Materials and Methods

### Poly (ADP-Ribose) Polymerase 1 KO Mice

The PARP1 KO mouse line 129S-*Parp1^TM 1*Zqw*^*/J^64^ was obtained from the Jackson Laboratory and maintained on a 129S1/SvImJ background. All animals were kept under a 12/12 h light/dark cycle and allowed food *ad libitum*. Animal procedures were reviewed and approved by the University of Michigan Institutional Animal Care and Use Committee. Embryonic dating was performed with vaginal plugging denoted as embryonic day 0.5 (E0.5). Following vaginal plugging, females were separated and sacrificed at the indicated time points.

### Cell Culture and Treatments

For primary NPC cell cultures, pregnant females were euthanized *via* cervical dislocation. Telencephalons were dissected from E14.5 embryos in ice-cold phosphate-buffered saline (PBS), meninges were removed, and cortices were dissociated into single cells with StemPro Accutase (ThermoFisher) for 5 min. NPCs were seeded as neurospheres in T75 flasks at 500,000 cells/mL and expanded for 2 days in NPC media (DMEM with GlutaMAX, 1% penicillin/streptomycin, and 2% B27 without RA) supplemented with epidermal growth factor (EGF, 20 ng/mL) and basic fibroblast growth factor (bFGF, 20 ng/mL) in a humidified 5% CO_2_/95% air incubator at 37°C. Half of the media was changed every day, with replenishment of EGF and bFGF each day. On day 3, neurospheres were dissociated with Accutase into a single cell suspension and plated in NPC media supplemented with bFGF (20 ng/mL). Plates were pre-prepared by incubating in Poly-L-Lysine (Sigma) 30 min or overnight followed by Fibronectin (1 μg/mL, Corning) for 2 h. Experiments on adherent NPCs were performed on day 2 after plating unless otherwise indicated.

To inhibit PARP1 enzymatic activity, NPCs were treated with Olaparib (a gift of C. Brenner) at the indicated concentrations (30, 50, or 100 nM) for 48 h starting on day 1 following plating. Half of the media was replenished each day, and cells were re-treated with Olaparib every 24 h. For Reelin-positive cell quantification, NPCs were plated on coverslips and treated with 50 nM Olaparib for 72 h. To test Olaparib efficacy, NPCs were pre-treated with 30, 50, or 100 nM Olaparib for 1 h prior to 10 min treatment with 50 μM H_2_O_2_. To assess RNA stability, NPCs were treated with Actinomycin D (10 μg/mL) for 2 or 4 h in the presence or absence of Olaparib (100 nM). HEK293T cells were maintained in DMEM with GlutaMAX, 1% penicillin/streptomycin, and 10% fetal bovine serum in a humidified 5% CO_2_/95% air incubator at 37°C.

### RNA-Sequencing and Analysis

Pregnant females were euthanized *via* cervical dislocation. E15.5 embryos were placed in ice-cold PBS, brains were dissected, and the cortical hemispheres were isolated. The meninges and ganglionic eminences were removed from the cortex, and the tissue was stored in RNAlater (Invitrogen) until RNA extraction. RNA was extracted from wild-type (WT) and PARP1 KO dorsal cortex using the Qiagen RNeasy Mini Kit with on-column DNase digestion (Qiagen), then analyzed with a BioAnalyzer to measure RNA quality. RNA with RNA integrity numbers (RINs) greater than 8 were sequenced (*n* = 4 of each genotype). Non-strand specific polyA-selected cDNA libraries were prepared. Single-end sequencing was then completed with read lengths of 50 nucleotides using an Illumina HiSeq-4000 Sequencing System. cDNA library preparation and sequencing were carried out by the University of Michigan DNA Sequencing Core. Sequences were mapped to the mouse genome (mm9) using HISAT, transcript counts obtained with HTseq-count, and differential gene expression analysis completed using DESeq2. All analysis was carried out with Galaxy^1^. The volcano plot was generated with RStudio. *p*-Values adjusted for multiple comparisons (*q*-value) < 0.05 indicated genes with statistically significant differences. Gene Ontology Analysis of dysregulated genes was performed using the Panther Classification system^2^. Protein interaction analysis was completed using Cytoscape software.

### Immunofluorescence and Quantification

Whole brains were dissected from E15.5 embryos or P0 pups in ice-cold PBS, fixed in 4% paraformaldehyde (PFA) in 1× PBS for 24 h, cryoprotected in 30% sucrose, embedded in OCT Compound (Fisher), and snap frozen in isopentane on dry ice. Frozen brains were cryosectioned at a thickness of 14 μm onto Superfrost plus slides (Fisher). Sections were blocked in 5% Normal Goat Serum (Jackson ImmunoResearch) with 2% Triton in PBS for 1 h, then incubated overnight at 4°C in primary antibody diluted in blocking buffer. The next day, sections were washed 3× in 1× PBS, incubated in corresponding Alexa-Fluor secondary antibodies (Invitrogen) diluted in blocking buffer (1:500) 1–2 h, and coverslipped with Fluoro-Gel II with DAPI (Electron Microscopy Services). Confocal *z*-stack images through the full depth of each section with a *z*-step of 2 μm were taken at a magnification of 20× or 40× with a Leica SP8 confocal microscope. For each biological replicate, cells from three sections and three images per section were quantified by an individual blind to the genotype and averaged. Cell number and layer thickness quantification were completed using Fiji software.

For Reelin NPC immunostaining, NPCs were plated on round glass coverslips. On day 2 after plating, coverslips were fixed in 4% PFA for 10 min, washed 3× in 1× PBS, then blocked in 10% Normal Goat Serum (Jackson ImmunoResearch) with 0.2% Triton in PBS for 1 h. Coverslips were then incubated in Reelin primary antibody overnight diluted in blocking buffer. Following three washes in 0.2% triton in PBS, coverslips were incubated in the corresponding Alexa-Fluor secondary antibody diluted in blocking buffer (1:500, Invitrogen) for 1–2 h, then mounted on slides with Fluoro-Gel II with DAPI (Electron Microscopy Services). For Reelin-expressing cell quantification in WT and PARP1 KO NPCs, images of whole coverslips were taken with a Leica SP8 confocal at 20× magnification with image stitching. For quantification of Reelin-expressing cells after PARP1 inhibition with Olaparib (50 μM) or shRNA-mediated knockdown, images of whole coverslips were taken with a Nikon TE300 inverted fluorescent microscope equipped with Stereoinvestigator (MBF Bioscience), using the slide scan module. For each biological replicate, all Reelin-expressing cells from three coverslips were quantified and averaged. Quantification and mean fluorescence intensity analysis were performed using Fiji. The following primary antibodies and concentrations were used in this study: mouse anti-Reelin (1:500, clone G10, Millipore #MAB5364), rabbit anti-TBR1 (1:250, Abcam #ab31940), and rat anti-CTIP2 (1:500, Abcam #ab18465).

### Cresyl Violet Staining and Brain Volume Quantification

Wild-type and PARP1 KO littermates were sacrificed at birth, and their brains were dissected then fixed in 4% PFA for 24 h. Brains were cryoprotected in 30% sucrose, embedded in OCT, then cryosectioned with a section thickness of 70 μm. Serial sections were stained with 0.5% cresyl violet using standard procedures. Slides were digitally scanned with the assistance of the University of Michigan *In Vivo* Animal Core (IVAC), and surface areas of each brain section were quantified with Fiji. To calculate the brain volume, the surface areas of each section were summed together and multiplied by the section thickness. Cortical surface area and thickness were quantified with Fiji.

### EdU Labeling *in vivo*

Pregnant dams from PARP1 heterozygous crosses were injected intraperitoneally with EdU (50 mg/kg) at E13.5. Littermate P0 pups were transcardially perfused at birth with PBS followed by 4% PFA, then whole brains were dissected and postfixed in 4% PFA for 24 h. Brains were then either cryoprotected in 30% sucrose, embedded in OCT, and cryosectioned at a thickness of 20 μm or embedded in 4% agarose and sectioned coronally on a vibratome at a thickness of 50 μm. EdU was visualized using the Click-iT EdU Cell Proliferation Kit (Invitrogen), following the manufacturer’s protocol. Brain sections were subsequently co-stained for the layer VI marker TBR1 (1:250) following the immunofluorescence staining protocol described above. Slides were coverslipped with Fluoro-Gel II containing DAPI (Electron Microscopy Services). Confocal *z*-stack images through the full depth each section were taken at 20× magnification with a Leica SP8 confocal microscope. Each image was separated into the area below layer VI, the area within layer VI, and the area above layer VI, and the number of EdU positive cells within each defined area was quantified using Fiji by an individual blinded to the genotype. For each biological replicate, three sections and three images per section were quantified and averaged.

### RNA Isolation and RT-qPCR

RNA was isolated from the cortex after removal of meninges and ganglionic eminences or from cultured NPCs using a Qiagen RNeasy Kit or ThermoFisher PureLink RNA Mini Kit, following the manufacturer’s instructions. RNA extraction from tissue was completed with on-column DNase digestion (Qiagen). Equal amounts of RNA (400 ng^–1^ μg) were reverse transcribed into cDNA using an iScript cDNA Synthesis Kit (Bio-Rad) following manufacturer’s instructions. cDNA was diluted fivefold for the PCR reaction. Quantitative PCR was performed using iTaq SYBR Green Supermix (Bio-Rad) with a Bio-Rad CFX96 Thermocycler. Each sample was run in duplicate. Each well of the 96-well plate contained 5 μL iTaq SYBR Green Supermix, 2.5 μL diluted cDNA, and 3 pmol of each forward and reverse primer. The cycling conditions were as follows: 95°C for 30 s followed by 39 cycles of 95°C for 5 s and 60°C for 30 s. Normalized Gene Expression (NGE) was calculated using the efficiency of each primer with the following formula: [efficiency_target^–CT^_target_/efficiency_reference^–CT^_ref_]. Fold changes in mRNA level were then calculated relative to controls for each experiment. All primer sequences are in Table 1.

**TABLE 1:** Sequences of primers used for quantitative RT-PCR.

Gene name	Forward sequence (5′ – 3′)	Reverse Sequence (5′ – 3′)
*Reln*	GTCGTGTCTTCTGGATCTTCTC	CAGCACTCTCTCCTCCTATCT
*Nav1*	CCAGCCACCAAGTTAGCAGA	CATGGGTGTCGCTGGAAGAT
*Tnc*	ACCATGCTGAGATAGATG TTCCAAA	CTTGACAGCAGAAACACCAATCC
*Txnip*	GTCAGTGTCCCTGGCTCCAAGA	AGCTCATCTCAGAGCTCGTCCG
Δ*Np73*	CTACCATGCTTTACGTCGG	CTGCCCATCTGGTCCAT
*TAp73*	GCACCTACTTTGACCTCCCC	GCACTGCTGAGCAAATTGAAC
*Car10*	GAGAGCAAGAGCCCAGAACTC	CTCACCAGTGGCAGAAATGGC
*Calb2*	CGGAGCTGGCGCAGAT	CTGCCTGAAGCACAAAAGGAA
*Parp1*	GGCAGCCTGATGTTGAGGT	GCGTACTCCGCTAAAAAGTCAC
*Gapdh*	TCACTGCCACCCAGAAGA	GCCAAGCCCTGAGCATAA
*Rpl19*	ACCTGGATGAGAAGGATGAG	ACCTTCAGGTACAGGCTGTG
β*-actin*	TCCCATTGAACACGGAGTG	CCTCGGTGAGAAGAATAGATGT

### shRNA-Mediated Poly (ADP-Ribose) Polymerase 1 Knockdown

Lentiviral packaging plasmids and scramble shRNA or *Parp1* shRNA constructs were transfected into HEK293T cells using Lipofectamine 3000 (Invitrogen). Lentiviral supernatants were collected and concentrated using Lenti-X Concentrator (Clontech) and titered with puromycin selection. To test the efficacy of PARP1 protein knockdown, NPCs were transduced with scramble or *Parp1* shRNA-expressing lentivirus at multiplicity of infection (MOI) 2 for 48 h. To assess the effects of PARP1 knockdown on gene expression, NPCs were transduced with scramble or *Parp1* shRNA-expressing lentivirus at MOI 3 for 48 h, then media was replaced with fresh NPC media and cells were lysed 24 h later. bFGF (20 ng/mL) was supplemented every 24 h. *Parp1* shRNA plasmid was obtained from Sigma (clone ID NM_007415.2-3021s21c1) with the following sequence: 5′-CCGGGAGTACATTGTCTACGACATTCTCGAGAATGTCGT AGACAATGTACTCTTTTTG-3′. Lentiviral packaging plasmids and scramble shRNA plasmid were gifts of S. Iwase.

### Luciferase Assay

Neural progenitor cells were co-transfected with a *Reln*-promoter luciferase construct that contained 2600 bp upstream of the *Reln* transcription start site (Chen et al., 2002) (a gift of D. Grayson) and *CMV*-Renilla at a 50:1 ratio with Lipofectamine 3000. NPCs were treated with either Olaparib (100 nM) for 24 or 48 h or Valproic Acid (VPA, 1 mM) for 12 or 24 h. Luciferase activity was measured using a Dual-Luciferase Assay Kit (Promega), following the manufacturer’s instructions. Luciferase intensity was measured with a BioTek plate reader. Final values were obtained by normalizing firefly luciferase to Renilla luciferase. Technical replicates were obtained in triplicate.

### Western Blot

For Reelin western blots, NPCs were lysed in RIPA buffer (Sigma) containing protease and phosphatase inhibitors. Equal amounts of protein were diluted in 4× Laemmli buffer with 10% β-mercaptoethanol (BME) and run on 4–15% SDS polyacrylamide gels. For western blotting of conditioned media, protein concentrations were quantified, and equal amounts of protein were diluted in 4× Laemmli buffer with 10% BME and run on 6% SDS polyacrylamide gels. Reelin in conditioned media was normalized to GAPDH in corresponding cell lysates. Gels were transferred onto PVDF membranes overnight at 4°C (wet transfer at 40 mA). Blots were subsequently blocked in Intercept Blocking Buffer (Licor) (for Reelin) or 5% Bovine Serum Albumin (BSA) (for GAPDH), incubated in primary antibody for 3 h, washed in 0.2% tween in PBS, then incubated in HRP-conjugated secondary antibody (Cell Signaling Technology). For pDab1 and PARP1 western blot, samples were lysed in RIPA buffer (Sigma) containing protease and phosphatase inhibitors. For PAR western blot, PARG inhibitor (1 μM ADP-HPD) was also added to RIPA lysis buffer. Equal amounts of protein were diluted in 4× Laemmli buffer with 10% BME and run on 7.5 or 8% SDS polyacrylamide gels, then transferred for 1.5 h onto PVDF membrane with a semi-dry transfer unit. Blots were then blocked in 5% BSA and immunoblotted for primary antibody overnight. The next day, blots were washed in 0.2% tween in PBS, then incubated in HRP-conjugated secondary antibody (Cell Signaling Technology). All blots were exposed with Pierce ECL Chemiluminescent Substrate and imaged on a Bio-Rad Chemidoc.

The following primary antibodies and concentrations were used: mouse anti-Reelin (1:2000, Millipore #MAB5364), mouse anti-PAR (1:1000, Trevigen #4335), mouse anti-GAPDH (1:2000, ThermoFisher #MA5-15738), rabbit anti-PARP1 (1:2000, Cell Signaling Technology #9532) and rabbit anti-pDab1 (1:1000, Cell Signaling Technology #3325). For all antibodies used, linearity was assessed, and the amount of total protein loaded onto gels was within the linear range of the antibody. Band densities were quantified using Image Lab software (Bio-Rad) and normalized to internal control GAPDH.

### Reelin Conditioned Media

HEK293T cells were transfected with pcDNA3 or Reelin ORF (10 kb)-expressing plasmids (D’Arcangelo et al., 1997) (Addgene plasmid #122443) with Lipofectamine 3000 (Invitrogen). Twenty-four hours after transfection, media was replaced with serum-free media. Twenty-four hours later, conditioned media was collected and stored at −80°C. For pDab1 induction, NPCs were treated with 200 or 400 μL conditioned media for 10 min. Conditioned media from WT and KO NPCs was collected from adherent cultures 48 h after plating. For induction of pDab1, protein secretion was blocked by pre-treating NPCs with Brefeldin A (BFA; 0.75 μg/mL, Sigma) for 3 h. NPCs were then treated with 2 mL conditioned media from WT NPCs (supplemented with 0.75 μg/mL BFA) for 20 min.

### Atomic Force Microscopy

Atomic force microscopy force-distance (F-D) measurements were performed using a TT-AFM (AFM Workshop, SC, United States) using contact mode AFM probes of 0.2 N/m nominal stiffness (PPP-CONTAuD from Nanosensors). Probes were functionalized by incubating the tip overnight at 4°C in 100 μg/mL N-cadherin. Immediately prior to measurements, probes were rinsed 4× in PBS. Probe stiffness was determined by the Sader method in air (Sader, 1998). The optical lever sensitivity was determined in fluid for each sample using the thermal noise method (Heim et al., 2004; Hutter, 2005).

Cells were cultured on round glass coverslips. Approximately 30 min prior to the beginning of measurements, a cell-coated coverslip was epoxied to an AFM stub and transferred to a fluid AFM imaging chamber with NPC media containing 10 mM HEPES and bFGF (20 ng/mL) and maintained at 37°C throughout measurements, which were taken within approximately 1 h for each sample. For conditioned media treatment, cells were treated with control or Reelin conditioned media for 30 min prior to the beginning of measurements. For each F-D measurement, the probe was engaged with the surface with approximately 9 nN force for 5 s before retracting at a rate of 2000 nm/s. F-D curves were evaluated using a custom Matlab script to objectively parse the maximum adhesion force developed, the adhesive force for stepwise detachments, and integrated work performed for each trace. These experiments were completed in collaboration with the Single Molecule Analysis in Real-Time (SMART) Center at the University of Michigan. Statistical outliers were excluded from the datasets [<Q1–1.5 × interquartile range (IQR) or >Q3 + 1.5 × IQR]. Statistical significance in average values was assessed *via* Mann–Whitney analysis and differences in histogram distributions were assessed with a Kolmogorov–Smirnov test.

### Statistics

All statistical analyses were performed using GraphPad Prism version 7.0, except for two-way ANOVA analysis of litter size, genotype, and P0 brain weight, which was performed using SAS in collaboration with the University of Michigan Center for Cancer Biostatistics. Bars for each of the graphs represent mean ± SEM. Legends for each figure contain statistical tests performed, number of biological replicates, and specific significance values.

## Results

### Poly (ADP-Ribose) Polymerase 1 KO Mice Have Brain Development Defects

**FIGURE 1:**
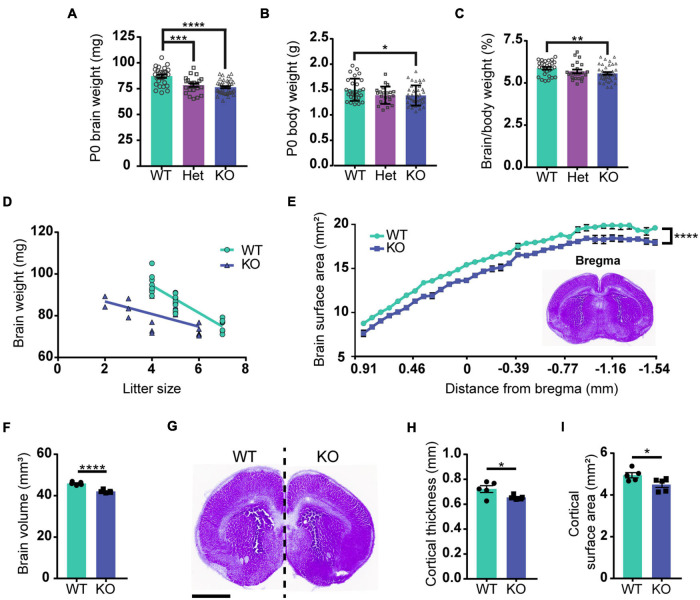
Loss of PARP1 results in reduced brain weight and cortical surface area at birth. **(A)** Brain weight of wild-type (WT) (*n* = 28), PARP1 heterozygous (Het) (*n* = 22), and PARP1 KO mice (*n* = 39) at birth (P0) shows a reduction in Het and KO mice compared with WT controls. ***p = 0.006; ****p < 0.0001 by Student’s unpaired *t*-test. **(B)** P0 body weight is reduced in KO mice compared to WT and Het animals. *p = 0.0315 by Student’s unpaired *t*-test. **(C)** The ratio of brain to body weight for each group shows a significant reduction in the relative size of KO brains compared to WT. **p = 0.0099 by Student’s unpaired *t*-test. **(D)** Litter size is inversely correlated with P0 brain weight. WT: *R*^2^ = 0.811, *p* < 0.0001; KO: *R*^2^ = 0.3725, *p* = 0.0043. *p*-Values indicate the significance of correlation within each genotype. Slopes are significantly different (*p* = 0.0029). Two-way ANOVA analysis indicated no significant interaction between litter size and genotype (*p* = 0.249). **(E)** Surface area measurement from rostral to caudal portions of the PARP1 KO brain indicates a reduced size throughout the rostral–caudal brain axis. The section designated as bregma is shown in the inset. Distance from bregma for the remaining sections is estimated. ****p < 0.0001 *via* two-way ANOVA. **(F)** Brain volume is reduced in PARP1 KO mice. **(G)** Representative image illustrating reduced brain surface area in PARP1 KO mouse. This section corresponds with bregma 1.045 mm in the adult brain. Scale bar, 1 mm. **(H)** PARP1 KO reduces cortical surface area and **(I)** cortical thickness. *p < 0.05; ****p < 0.0001 by Student’s unpaired *t*-test. Quantification shown represents brain slices corresponding with bregma 1.045 mm in the adult mouse brain. **(E–I)** *n* = 5 WT and 5 KO brains from 4 L.

Previous studies reported that brain size is reduced in PARP1 KO mice from P11 to adulthood (Plane et al., 2012; Hong et al., 2019). We now find that both body and brain weights are smaller in PARP1 KO mice at birth (Figures 1A,B), but that brain weight is reduced to a larger relative extent than the mass of the entire body (Figure 1C). While there is an inverse correlation between litter size and brain weight in WT mice, two-way ANOVA analysis showed no significant interaction between genotype and litter size (Figure 1D), indicating that decreased brain weight in PARP1 KO animals is driven exclusively by genotype. To assess whether decreased brain weight is due to a reduction in brain size in a specific area, we collected serial coronal sections from WT and PARP1 KO P0 brains, measuring the surface area of each section on the rostral–caudal axis. We found that the surface area of PARP1 KO brain sections tends to be smaller than their WT littermates at each level along the rostral–caudal axis, indicating that brain size is reduced overall rather than in a specific area (Figure 1E), which corresponds with a reduction in brain volume in PARP1 KO animals (Figure 1F). Furthermore, the decreased brain size in PARP1 KO mice is associated with a reduced cortical surface area and thickness (Figures 1G–I).

**FIGURE 2:**
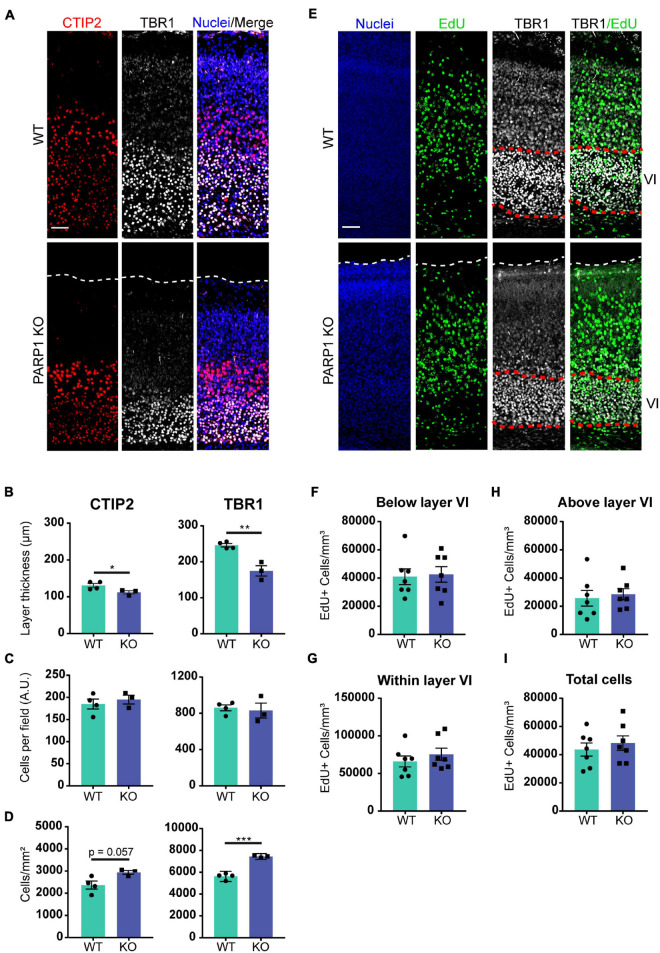
Poly (ADP-ribose) polymerase 1 KO mice have defects in cortical development. **(A)** Representative images from CTIP2+ (red, layer V) and TBR1+ (white, layer VI) cells in the cortex of P0 mice. Nuclei were labeled with DAPI (blue). Scale bar, 50 μm. **(B)** The thickness of TBR1 and CTIP2-expressing cell layers is decreased in KO P0 brains. *p = 0.0498, **p = 0.0026 by Student’s unpaired *t*-test. **(C)** The number of TBR1 and CTIP2 positive cells per field in P0 brains does not differ between genotypes. **(D)** The density of CTIP2+ and TBR1+ cells is increased in KO P0 brains. ***p = 0.0002 by Student’s unpaired *t*-test. **(A–D)** *n* = 4 WT and 3 KO animals from 2 L, average of three sections in the somatosensory cortex region per animal and three images per section. **(E)** Representative images of coronal cortical sections at P0 of mice in which proliferating cells (green) were labeled by injecting EdU (IP, 50 mg/kg) to the dam at day 13.5 of pregnancy. Nuclei were labeled with DAPI (blue), and images were co-stained with layer VI marker TBR1 (white). Scale bar, 50 μm. **(F)** PARP1 loss does not alter the number of EdU-labeled cells below layer VI, **(G)** within layer VI, or **(H)** above layer VI. **(I)** PARP1 loss does not alter the total number of EdU-labeled cells at E13.5 **(E–I)** *n* = 7 animals per genotype, average of three sections in the somatosensory cortex region per animal and three images per section.

The findings described above suggest that PARP1 loss alters brain development. One of the key events in neurodevelopment is the inside-out migration of neuronal progenitors, whereby early-born neurons form deeper brain layers, while later-born neurons form superficial layers (Jiang and Nardelli, 2016). In concordance with the reduction in cortical thickness at birth, immunostaining for markers of layer V and layer VI neurons (CTIP2 and TBR1 respectively; Figure 2A), showed that these layers are also thinner in PARP1 KO mice at birth (Figure 2B). However, this reduction does not correspond with an altered number of neurons within each layer (Figure 2C), indicating that the loss of PARP1 results in a normal number of more densely packed neurons (Figure 2D). Accordingly, labeling of early-born neurons *via* EdU injection at E13.5 showed no differences in placement of these neurons in layer VI at birth, nor was there any difference in the total number of EdU-labeled cells between genotypes (Figures 2E–I). Together, these results suggest that reduced brain weight in PARP1 KO mice reflects decreased cortical thickness and increased cell density without altering progenitor cell quantity or migration patterns.

### Loss of Poly (ADP-Ribose) Polymerase 1 Increases the Expression Levels of Genes Associated With Cell Migration and Adhesion in the E15.5 Cortex

**FIGURE 3:**
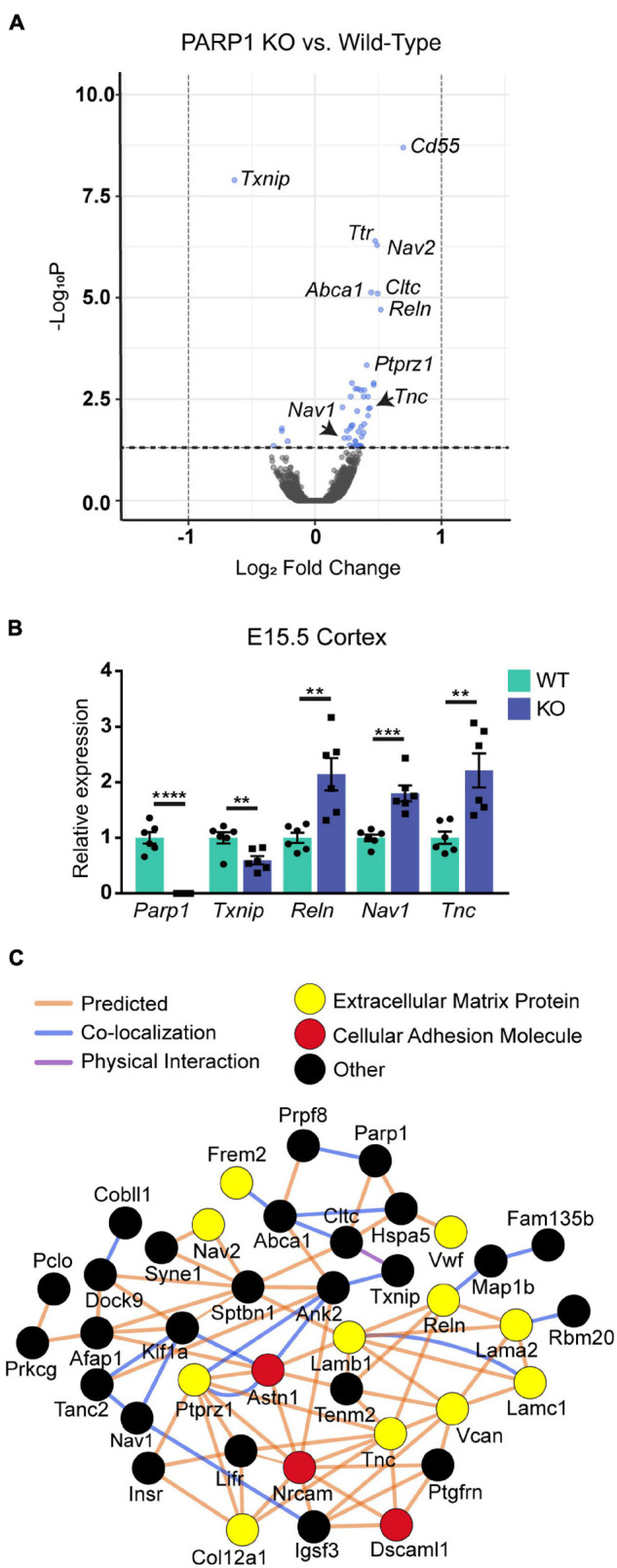
Expression levels of genes associated with neuronal migration and cell adhesion are increased at E15.5 in the cortex of PARP1 KOs. **(A)** Volcano plot of the differentially expressed genes in the PARP1 KO cortex at E15.5 identified by RNA-sequencing (*n* = 4 biological replicates per genotype). Genes with significantly altered expression are indicated in blue (*q*-value < 0.05). Upregulated genes comprised 88% (42/48 total) of all genes exhibiting significant differential expression in KO embryonic cortex. **(B)** RT-qPCR validation of a subset of differentially expressed genes (*n* = 6 of each genotype). Levels of target mRNAs were normalized to β*-actin* levels. **p < 0.01; ***p < 0.001; ****p < 0.0001 by Student’s unpaired *t*-test. **(C)** Interactions among proteins encoded by differentially expressed genes were identified with Cytoscape software based upon bioinformatic prediction (orange lines), co-localization (blue lines), or physical interaction (purple lines). Proteins involved in the extracellular matrix (yellow) and cell adhesion (red) are highlighted. All others are colored in black.

To determine if PARP1 loss alters the brain transcriptome, we performed RNA-sequencing of the E15.5 WT and KO cortex. We identified 48 genes whose levels are significantly altered by PARP1 loss-of-function. Remarkably, in contrast to reports that PARP1 promotes transcription in neuronal cells (Tapia-Páez et al., 2008; Hau et al., 2017; Azad et al., 2018), most of the changes in expression in the brains of PARP1 KOs reflect increases in mRNA levels (Figure 3A). RT-qPCR validated the altered expression of *Reln*, *Nav1*, *Tnc*, and *Txnip* in the embryonic cortex (Figure 3B). Furthermore, a subset of these genes (*Tnc* and *Reln*) continues to be increased to a similar extent in the P0 cortex of PARP1 KO mice (data not shown). The genes altered by PARP1 loss encode proteins involved in various processes, with a particular enrichment for cell adhesion, axon development, dendrite development/morphogenesis, and cell migration (Table 2). Similarly, molecular interaction network analysis of the differentially expressed genes suggest direct interactions among proteins which comprise parts of the brain extracellular matrix (ECM) (Figure 3C), an important component in the regulation of neuronal migration and lamination (Franco and Müller, 2011), including Laminins (*Lamb1*, *Lama2*, and *Lamc1*), Reelin (*Reln*), Tenascin C (*Tnc*), Versican (*Vcan*), and Phosphacan (*Ptprz1*). These proteins in turn interact with Collagen Type XII Alpha 1 Chain (*Col12a1*) and several cell adhesion molecules (*Astn1*, *Nrcam*, and *Dscaml1*). Taken together, these findings suggest that the brain defects in neonatal PARP1 KO mice could result from alterations in ECM function.

**TABLE 2:** Poly (ADP-ribose) polymerase 1 loss upregulates the expression of genes associated with cell adhesion, axon development, dendrite morphogenesis, and cell migration in the E15.5 cortex.

GO biological process	Fold enrichment	False discovery rate
Cell adhesion (GO:0007155)	7.95	0.00001
Cell morphogenesis involved in differentiation (GO:0000904)	8.51	0.0002
Axon development (GO:0061564)	10.38	0.00072
Regulation of dendrite morphogenesis (GO:0048814)	20.51	0.00293
Neuron projection morphogenesis (GO:0048812)	8.24	0.00302
Plasma membrane bounded cell projection morphogenesis (GO:0120039)	8.15	0.00304
Cell projection morphogenesis (GO:0048858)	8.05	0.00322
Axonogenesis (GO:0007409)	9.91	0.00327
Regulation of cell morphogenesis involved in differentiation (GO:0010769)	9.74	0.00355
Regulation of dendrite development (GO:0050773)	12.47	0.0206
Establishment of organelle localization (GO:0051656)	8.77	0.0235
Cerebral cortex radially oriented cell migration (GO:0021799)	39.25	0.0276
Axon guidance (GO:0007411)	11.05	0.0336
Neuron projection guidance (GO:0097485)	10.95	0.0343
Glial cell migration (GO:0008347)	34.57	0.0361
Gliogenesis (GO:0042063)	10.25	0.041

### Loss of Poly (ADP-Ribose) Polymerase 1 Results in Increased Number of Cajal–Retzius Cells in the Cortex

**FIGURE 4:**
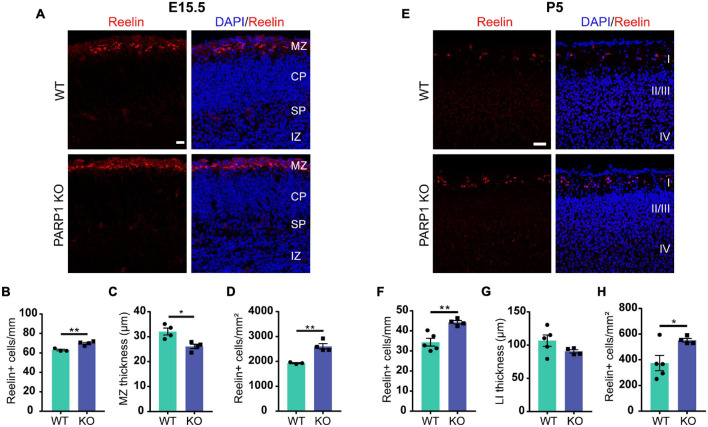
Loss of PARP1 increases the number of Cajal–Retzius cells in the E15.5 and P5 cortex. **(A)** Representative images of coronal sections of brain cortex from E15.5 WT and PARP1 KO embryos following Reelin immunostaining (red). Nuclei are labeled with DAPI (blue). Scale bar, 20 μm. Marginal zone (MZ), cortical plate (CP), subplate (SP), and intermediate zone (IZ) are labeled. MZ is defined as area containing diffuse Reelin staining. **(B)** The number of Reelin-expressing cells along the marginal zone is increased, **(C)** marginal zone thickness is decreased, and **(D)** Reelin+ cell density is increased in the KO E15.5 brain (*n* = 4 for each genotype from 7 L, three sections per animal including rostral, medial, and caudal regions, and three images per section). **p < 0.01 by Student’s unpaired *t*-test. **(E)** Representative images of coronal sections of cortex from P5 WT and PARP1 KO embryos showing Reelin immunostaining in red. Nuclei are labeled with DAPI (blue). Scale bar, 50 μm. **(F)** The number of Reelin-expressing cells in layer I is increased, **(G)** layer I (LI) thickness shows a decreasing trend, and **(H)** density of Reelin-expressing cells in layer I is increased in the P5 KO brain (*n* = 4 WT and 5 KO from 8 L, three sections per animal from rostral, medial, and caudal brain regions, and three images per section). *p < 0.05 and **p < 0.01 by Student’s unpaired *t*-test. *p* = 0.15 for P5 LI thickness.

*Reln* encodes the glycoprotein Reelin, which is specifically expressed by CR cells within the developing brain’s marginal zone (Ogawa et al., 1995). Reelin is critical for cortical layering and neuronal migration, and mice lacking Reelin (known as *Reeler*) have severely malformed brains and die prematurely (Meier and Hoag, 1962; Hamburgh, 1963; Goffinet et al., 1984). Quantification of Reelin-expressing cells in the E15.5 brain revealed an increased number of CR cells along the marginal zone and a reduction in marginal zone thickness in PARP1 KO brains, resulting in increased density of CR cells within the marginal zone (Figures 4A–D). A similar increase in CR cell density was observed at P5 (Figures 4E–H), whereas no CR cells were detected at P21 in both WTs and mutants (data not shown). Together, these results suggest that PARP1 regulates CR cell generation and does not affect their programmed cell death, which begins around P8 in WT mice (del Río et al., 1995).

### Loss of Poly (ADP-Ribose) Polymerase 1 Increases Levels of Genes Expressed by Cajal–Retzius Cells and Reelin Protein in Neural Progenitor Cells

**FIGURE 5:**
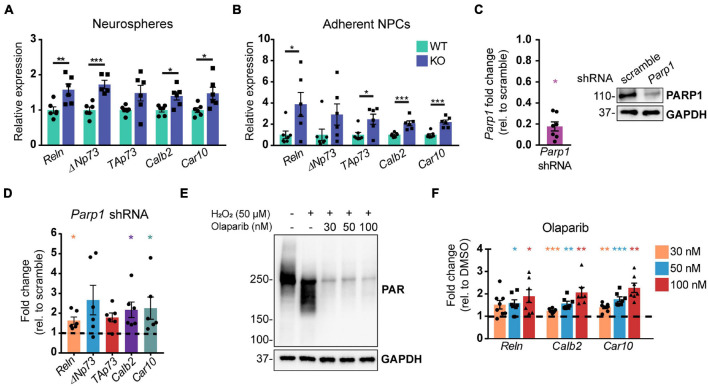
Poly (ADP-ribose) polymerase 1 loss by KO, shRNA knockdown, or pharmacological inhibition increases mRNA levels of genes expressed by Cajal–Retzius cells. **(A,B)** Quantitative RT-PCR for genes expressed by CR cells in neurospheres **(A)** and adherent NPCs **(B)** from WT and KO cultures (*n* = 6 of each genotype) demonstrate increased expression of genes expressed by CR cells. Target mRNA levels were normalized to *Rpl19*. *p < 0.05; **p < 0.01; ***p < 0.001 by Student’s unpaired *t*-test. Relative WT versus KO expression of *TAp73* in **(A)** is *p* = 0.06 and of Δ*Np73* in **(B)** is *p* = 0.01. **(C)** *Parp1* transcript (left) and protein (right) levels are substantially reduced following transduction of wild-type NPCs with *Parp1* shRNA expressing lentivirus. Fold change in transcript levels was calculated relative to scramble (scr) shRNA control (*n* = 7). *Parp1* expression was normalized to *Gapdh*. *p = 0.0156 by Wilcoxon test. **(D)** mRNA transcripts expressed by CR cells are increased after wild-type NPC transduction with *Parp1* shRNA-expressing lentivirus (MOI 3) for 72 h. Gene expression was normalized to *Gapdh.* Fold change relative to scramble (scr) shRNA-transduced NPCs is plotted (*n* = 6); Δ*Np73* and *TAp73*: *p* = 0.06; *p = 0.0313 by Wilcoxon test. **(E)** PAR western blot of wild-type NPCs after Olaparib pre-treatment (at indicated concentrations) for 1 h followed by 10 min H_2_O_2_ treatment (50 μM) shows that Olaparib inhibits H_2_O_2_-induced PARylation. **(F)** Olaparib-mediated inhibition of PARP1 in wild-type NPCs for 48 h increases mRNA levels of genes expressed by CR cells. Gene expression was normalized to *Rpl19*. Fold changes were calculated relative to DMSO control treated cells (*n* = 7–8). *Reln* (30 nM): *p* = 0.056; *p < 0.05; **p < 0.01; ***p < 0.001 by repeated measures one-way ANOVA.

To interrogate the mechanism by which PARP1 regulates CR cell numbers, we analyzed telencephalon-derived NPCs in culture. RT-qPCR demonstrated that neurospheres and adherent NPCs from PARP1 KO mice have increased mRNA levels of several genes expressed by CR cells, including *Reln*, the *Trp73* isoforms *TAp73* and Δ*Np73*, as well as *Car10* and *Calb2* (Yamazaki et al., 2004; Figures 5A,B). To assess whether these gene changes are specifically associated with PARP1 loss, we knocked down PARP1 in WT NPCs *via* transduction of a *Parp1* shRNA-expressing lentivirus (Figure 5C). As observed in PARP1 KO NPCs, acute PARP1 knockdown led to a significant increase in *Reln*, *Calb2*, and *Car10*, with a trend of increased *Trp73* isoforms (Figure 5D). Furthermore, treatment with the PARP1 inhibitor Olaparib, which blocks PARylation (Figure 5E), had similar effects on the expression of *Reln, Car10*, and *Calb2* in WT NPCs (Figure 5F). These results indicate that PARP1 PARylation influences the mRNA levels of genes expressed by CR cells.

### Poly (ADP-Ribose) Polymerase 1 Loss of Function Increases Cajal–Retzius Cell Abundance in Neural Progenitor Cell Cultures

**FIGURE 6:**
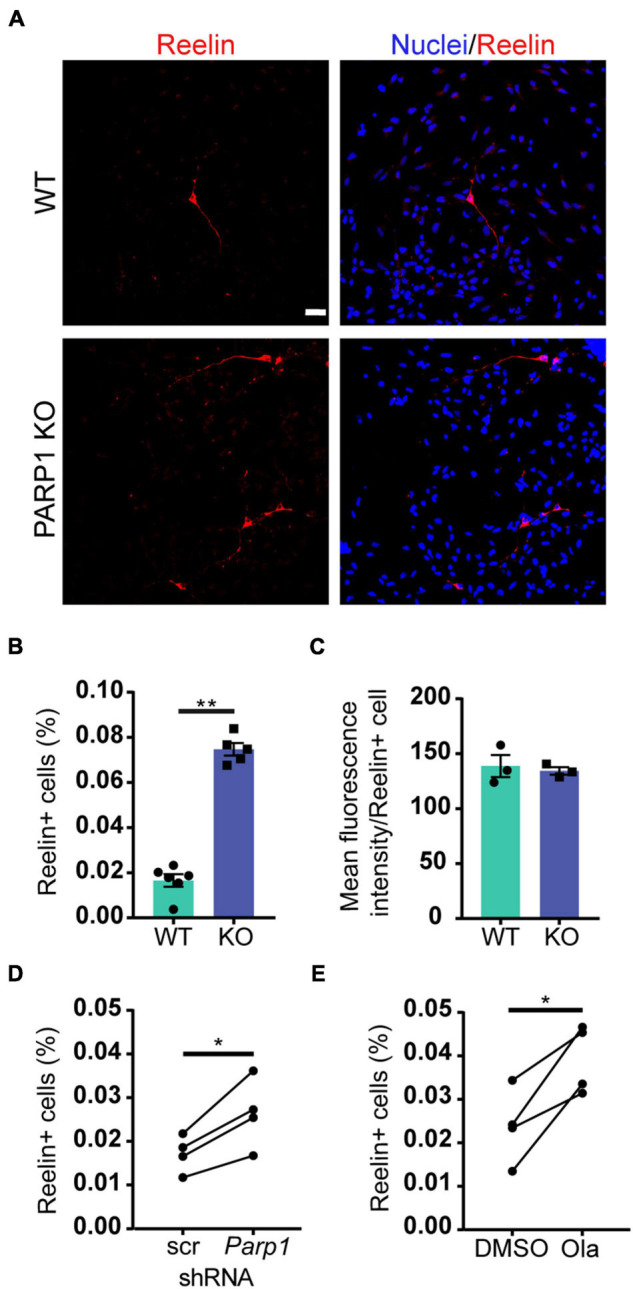
Poly (ADP-ribose) polymerase 1 loss increases CR cell abundance in NPC cultures. **(A)** Immunostaining for Reelin (red) demonstrates presence of Reelin-expressing cells in WT and PARP1 KO NPC cultures. Nuclei were labeled with DAPI (blue). Scale bar, 20 μm. **(B)** Quantification of the percentage of Reelin-expressing cells within NPC cultures shows increased proportion of Reelin+ cells in KO cultures (*n* = 6 WT and 5 KO biological replicates and 3 coverslips per replicate). **p = 0.0013 by Mann–Whitney test. **(C)** The mean fluorescence intensity of Reelin expression per cell does not differ between genotypes. Each point on graph represents average Reelin intensity per cell from three biological replicates of each genotype. **(D)** The percentage of Reelin-expressing cells in wild-type NPC cultures increases after shRNA-mediated PARP1 knockdown for 72 h (*n* = 4 biological replicates per group and 3 coverslips per replicate). *p = 0.0174 by Student’s paired *t*-test. **(E)** The percentage of Reelin-expressing cells in wild-type NPC cultures increases with PARP1 inhibition *via* Olaparib (50 nM) for 72 h. Olaparib was re-treated every 24 h (*n* = 4 biological replicates per group and 3 coverslips per replicate). *p = 0.022 by Student’s paired *t*-test.

**FIGURE 7:**
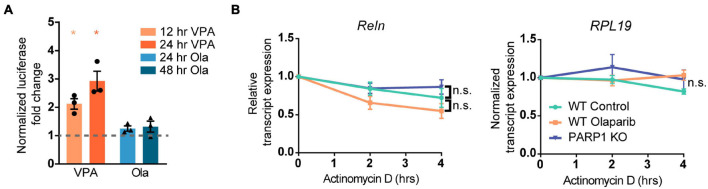
Poly (ADP-ribose) polymerase 1 loss of function does not alter the activity of the *Reln* promoter or the stability of the *Reln* transcript. **(A)** NPCs transfected with plasmids expressing the putative *Reln* promoter (2600 base pairs upstream of the transcription start site) upstream of firefly luciferase did not show increased luciferase activity after treatment with Olaparib (Ola, 100 nM) for 24 or 48 h. Valproic Acid (VPA) is a known positive regulator of the *Reln* transcript and was used as a positive control. *p < 0.05 by one-way ANOVA. **(B)** PARP1 KO or PARP1 inhibition with Olaparib (100 nM) does not alter the stability of the *Reln* transcript. RNA polymerase II was inhibited by treatment with Actinomycin D (10 μg/mL) for 2 and 4 h and transcript levels were assessed. *Reln* was normalized to *RPL19* expression, which did not decline over 4 h. No significant difference was observed between slopes for any of the groups.

To explore if the changes in Reelin levels in tissue culture reflect differences in levels of Reelin expression per cell or, alternatively, in the number of Reelin expressing cells, we immunostained adherent NPCs for Reelin. Cultures of PARP1 KO NPCs contained a higher proportion of Reelin-positive cells without changes in the intensity of Reelin staining per cell (Figures 6A–C). Similarly, shRNA-mediated PARP1 knockdown and pharmacological PARP1 inhibition increased the proportion of Reelin-positive cells in WT NPC cultures (Figures 6D,E). These results suggest that PARP1 and its enzymatic function are critical for regulating CR-like cell abundance *in vitro* rather than having a direct transcriptional effect on gene expression in existing CR cells. This finding is consistent with results from *in situ* hybridization showing that the number of *Reln* transcripts per CR cell in the E15.5 brain is not altered after PARP1 loss (data not shown). Mechanistically, this finding is further supported by the observation that the activity of a firefly luciferase *Reln* promoter reporter construct (Chen et al., 2002) transfected into WT NPCs is not altered by PARP1 inhibition (Figure 7A). In addition, we observed no alteration in *Reln* mRNA stability in PARP1 KO NPCs (Figure 7B) and found no evidence of PARP1 binding to the *Reln* gene or its mRNA *via* chromatin or RNA immunoprecipitation, respectively (data not shown). Together, these results suggest that PARP1 regulates the abundance of Reelin-expressing cells *in vivo* and *in vitro* and that this function of PARP1 is not mediated by direct transcriptional regulation of the *Reln* gene.

### Medium Conditioned by Neural Progenitor Cells Contains Bioactive Reelin

**FIGURE 8:**
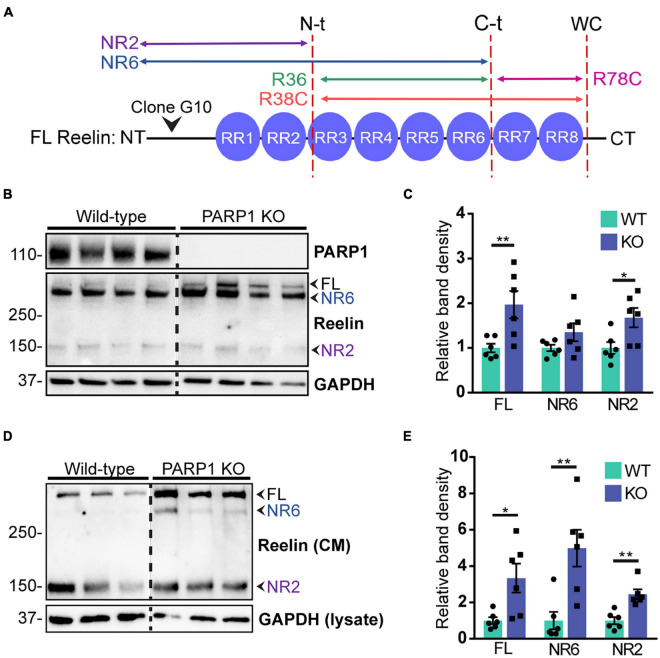
Poly (ADP-ribose) polymerase 1 KO NPC cultures overexpress Reelin protein. **(A)** Schematic drawing of Reelin depicting its proteolytic cleavage sites (vertical dotted lines) and the site of recognition for the N-t clone G10 Reelin antibody. This antibody recognizes full-length (FL) Reelin as well as the NR6 and NR2 Reelin fragments. **(B)** Reelin western blot from WT and PARP1 KO NPC lysates shows increased Reelin protein in KO NPC cultures. Arrows indicate the FL, NR2, and NR6 fragments. **(C)** Quantification of relative band density of FL Reelin, the NR2 fragment, and the NR6 fragment normalized to GAPDH (*n* = 6). NR6: *p* = 0.24; *p = 0.026; **p = 0.0087 by Mann–Whitney test. **(D)** Western blot for Reelin in conditioned media (CM) and GAPDH in cell lysates of WT and KO NPCs. **(E)** Quantification of band densities of FL Reelin, NR2, and NR6 fragments normalized to GAPDH in cell lysates (*n* = 6 separate cultures) indicate increased Reelin present in media conditioned by KO NPC cultures. *p < 0.05; **p < 0.01 by Mann–Whitney test.

CR cells regulate neuronal migration in part through secretion of Reelin, which binds to its receptors Apolipoprotein E Receptor 2 (ApoER2) and Very Low Density Lipoprotein Receptor (VLDLR) on nearby migrating neurons (Hirota and Nakajima, 2017). Reelin is proteolytically cleaved at three sites, resulting in multiple protein fragments (Lambert de Rouvroit et al., 1999; Krstic et al., 2012; Figure 8A). Full-length Reelin is hypothesized to be the most catalytically active, whereas N-terminal (N-t) cleavage (Kohno et al., 2009; Ogino et al., 2017) and loss of the C-terminal region of Reelin (Nakano et al., 2007; Kohno et al., 2015) reduce its catalytic activity. To identify the Reelin fragments expressed in NPC cultures, we immunoblotted NPC lysates and conditioned media for Reelin using an N-t antibody. Analysis of cell lysates demonstrated a significant upregulation in the 430 kDa full-length Reelin and the 160 kDa NR2 fragment in PARP1 KO NPCs (Figures 8B,C). Similarly, western blotting of conditioned media collected from NPCs showed increased immunoreactivity for the full length Reelin and the NR6 and NR2 fragments, indicating increased Reelin secretion in PARP1 KO NPC cultures (Figures 8D,E).

**FIGURE 9:**
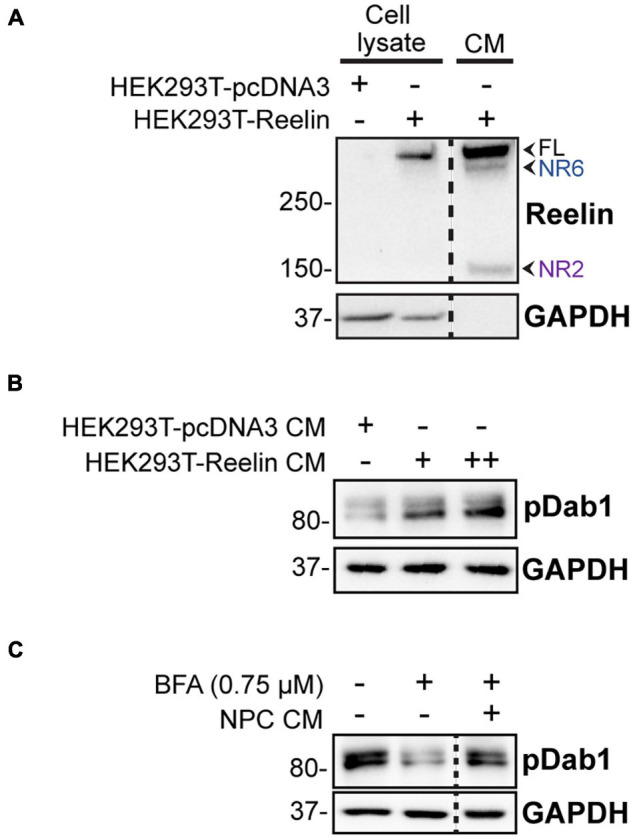
Reelin induces Dab1 phosphorylation in NPCs. **(A)** Western blot of medium conditioned by HEK293T cells transfected with a Reelin-expressing plasmid or empty vector (pcDNA3) and their cell lysates indicates expression and secretion of Reelin. Lysates and conditioned medium (CM) were collected 24 h after transfection. Arrows indicate full-length (FL) Reelin and NR2 and NR6 fragments. **(B)** WT NPCs treated with 200 μL (+) or 400 μL (++) conditioned medium (CM) from Reelin-transfected HEK293T cells for 10 min have increased Dab1 phosphorylation (pDab1). **(C)** A total of 20 min treatment with CM from WT NPC cultures increases Dab1 phosphorylation in WT NPCs (*n* = 4 separate cultures). Pretreatment with Brefeldin A (BFA; 0.75 μg/mL) for 3 h prior to CM treatment reduces Dab1 phosphorylation in WT NPCs, indicating that the state of Dab1 phosphorylation depends on the ability of cells to secrete proteins. Addition of BFA to CM after harvesting does not affect pDab1 induction. Relative band density was normalized to GAPDH. Fold change was 2.19 with *p* = 0.023 by one-sample *t*-test.

To determine the Reelin present in the NPC conditioned medium is bioactive, we tested if it induces phosphorylation of Dab1, a key step in the intracellular signaling cascades initiated by Reelin (Hiesberger et al., 1999; Lambert de Rouvroit et al., 1999). As a positive control, we used medium conditioned by HEK293T cells transfected with a Reelin expression plasmid, which clearly induced Dab1 phosphorylation in NPCs (Figures 9A,B). To test if NPC conditioned media also induces Dab1 phosphorylation in NPCs, we had to block NPC protein secretion with BFA to prevent autocrine Reelin stimulation. Exposure of BFA treated NPCs to media conditioned by WT NPCs also induced Dab1 phosphorylation (Figure 9C), indicating that Reelin secreted by CR cells in culture has physiological effects on NPCs and suggesting that excess secreted Reelin due to PARP1 loss might alter downstream functions associated with Reelin signaling.

### Poly (ADP-Ribose) Polymerase 1 Loss Increases Neural Progenitor Cells Adhesiveness to N-Cadherin

**FIGURE 10:**
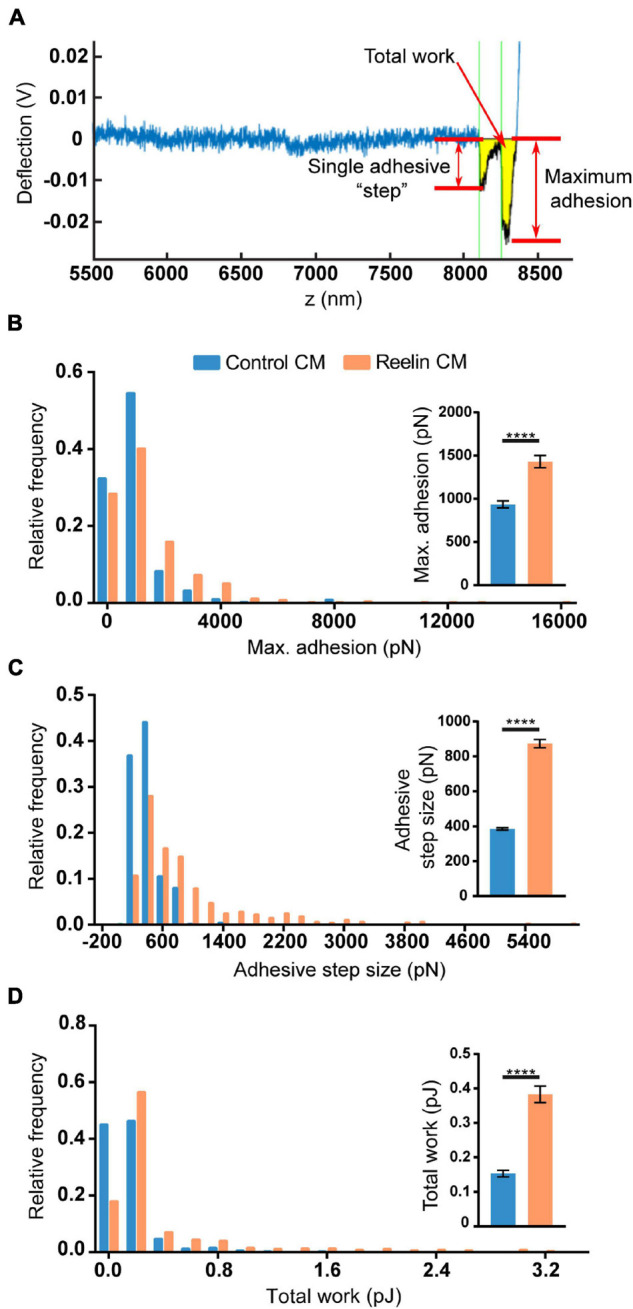
Reelin increases NPC adhesiveness to N-cadherin. **(A)** Example trace generated by AFM from an N-cadherin-coated cantilever in contact with a single cell. From each trace, adhesive step size, maximum adhesion, and total work (yellow shading) were calculated. Thirty-minute treatment of WT NPCs with conditioned media (CM) from Reelin-transfected HEK293T cells increases **(B)** maximum adhesion, **(C)** adhesive step size, and **(D)** total work compared to CM from pcDNA3-transfected HEK293T cells (control CM) (*n* = 402–536 from two biological replicates). Distributions are significantly different (*p* < 0.0001 by Kolmogorov–Smirnov test). Inset graphs show average values. ****p < 0.0001 by Mann–Whitney test.

**FIGURE 11:**
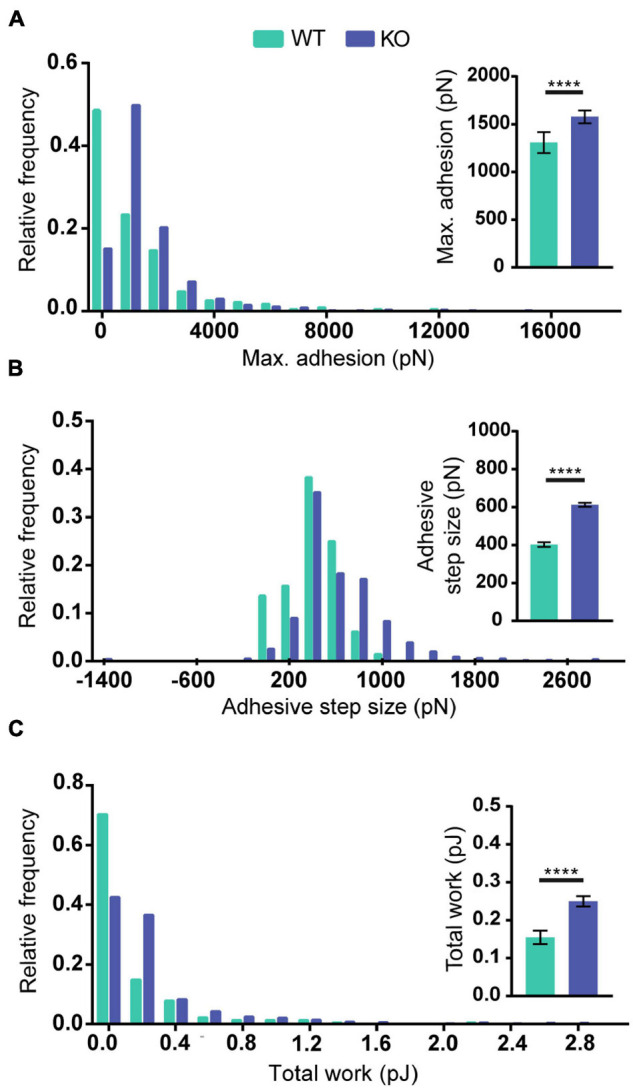
Poly (ADP-ribose) polymerase 1 loss increases NPC adhesiveness to N-cadherin. Maximum adhesion **(A)**, adhesive step size **(B)**, and total work **(C)** are higher in NPCs from KOs than from WTs (*n* = 229–742 from 4 WT and 5 KO biological replicates). Distributions are significantly different (*p* < 0.0001 by Kolmogorov–Smirnov test). Inset graphs show average values. ****p < 0.0001 by Mann–Whitney test.

To test if the increased levels of Reelin present in media conditioned by PARP1 KO NPCs has functional consequences, we focused on cell adhesion since a prior study showed that Reelin increases neuronal adhesion to N-cadherin using AFM (Matsunaga et al., 2017). Using a similar approach (Figure 10A), we found that treatment with medium conditioned by Reelin-transfected HEK293T cells increases adhesion of N-cadherin in WT NPCs (Figures 10B–D). Furthermore, we found that adhesion to N-cadherin is increased in PARP1 KO NPCs (Figure 11), suggesting that the increased Reelin levels alter the adhesive state of PARP1 KO cells.

## Discussion

Our findings uncover a new role for PARP1, i.e., the regulation of CR cell development. Loss of PARP1 leads to increases in the number of Reelin-expressing cells in the cortex *in vivo* and in cultured NPCs. While relative increases in *Reln* transcript levels were demonstrated in bulk RNA from the brain of PARP1 KOs, we did not detect any effects of PARP1 loss-of-function on *Reln* transcript levels per CR cell *in vivo* or identify any evidence of PARP1 binding to the *Reln* gene or its mRNA to directly influence *Reln* transcription in cultured cells. These results suggest that PARP1 influences CR cell generation rather than more directly impacting the expression of CR cell genes. The mechanism by which this occurs is unclear, but the overabundance of CR cells in PARP1 KO brains at E15.5, together with the fact that these cells do not undergo programmed cell death until P8 in WT mice (del Río et al., 1995), indicate that PARP1 does not influence the number of CR cells by regulating the time-course of apoptosis. Accordingly, we did not observe any remaining CR cells in the PARP1 KO cortex at P21. A potential mechanism by which PARP1 could regulate CR cell generation is through miRNAs. PARP1 has been shown to regulate the expression of a number of miRNAs (Chacon-Cabrera et al., 2015; Nozaki et al., 2018; Wang et al., 2019), and disruption of miRNA biogenesis through Dicer depletion in Nestin-expressing cells causes a similar increase in CR cell abundance and *Reln* expression (McLoughlin et al., 2012). Similar to findings in PARP1 KOs (Hong et al., 2019), McLoughlin et al. (2012) reported that Dicer depletion reduces cell proliferation and increases apoptosis in the E15.5 cortex. Additionally, PARP1 has a number of known transcription factor targets that have been shown to regulate CR progenitor cell development and migration, including Ascl1/Mash1, Pax6, and Cxcl12 (Stoykova et al., 2003; Ju et al., 2004; Borrell and Marín, 2006; Dixit et al., 2011, p. 1; Yoo et al., 2011; Marković et al., 2013; Tolić et al., 2019; Kaddour et al., 2020). Further studies will be necessary to test the links between PARP1, transcription factor activity, miRNAs, and CR cell generation in the embryonic brain.

Cajal–Retzius progenitor cells migrate tangentially into the cortex from distinct areas of the developing brain, including the cortical hem, pallial septum, and the pallial-subpallial boundary (Barber and Pierani, 2016). Interestingly, CR cells from each of these origins express different combinations of the proteins Reelin, Calretinin, and p73, which makes it possible to identify subpopulations of CR cells in the developing cortex (Bielle et al., 2005). Additionally, CR cell progenitors from each of these domains migrate to different areas of the cortex on the dorsal–ventral and rostral–caudal axes (Barber and Pierani, 2016). Our experiments demonstrate increased mRNA levels of each of these three associated proteins in PARP1 KO NPC cultures and increased CR cell abundance in multiple areas of the cortex on the rostral–caudal axis, suggesting that PARP1 does not affect the migration of one CR progenitor cell subpopulation. However, further studies analyzing the distribution of CR cells along the dorsal–ventral axis in addition to the rostral–caudal axis are necessary to confirm this hypothesis.

In contrast to PARP1 KOs, in which we found the number of Reelin-positive cells to be increased in the marginal zone, its normal site of expression, previous studies explored the effects of ectopic Reelin expression during embryonic development. The Nakajima group tested the effects of ectopic Reelin expression in deeper cortical layers using *in utero* electroporation of a Reelin-expressing plasmid into the lateral ventricle of E14.5 embryos (Kubo et al., 2010; Matsunaga et al., 2017). In line with our findings that Reelin overexpression is associated with increased NPC adhesion, this ectopic Reelin expression induced the formation of aberrant neuronal aggregates that appeared to be mediated by N-cadherin-dependent neuronal adhesion. In a transgenic mouse line with Nestin-driven Reelin expression, ectopic Reelin expression in ventricular and subventricular zone NPCs was shown to increase NPC proliferation, possibly through alterations in Notch signaling, and to result in an increased number of TBR1, TBR2, and CTIP2-positive neurons (Lakoma et al., 2011). These findings contrast with previous observations in PARP1 KOs of decreased NPC proliferation (Hong et al., 2019) and our observation of increased TBR1 and CTIP2-positive neuronal density without changes in cell number. Taken together, these findings highlight the different consequences that Reelin overexpression has on brain development depending upon the timing, location, and nature of its overexpression.

Reelin influences cell migration in part through regulating cell adhesion *via* integrin (Sekine et al., 2012), L1 (Lutz et al., 2017), nectins (Gil-Sanz et al., 2013), and N-cadherin (Franco et al., 2011; Matsunaga et al., 2017). In accordance with this, we found that Reelin treatment and PARP1 loss increase NPC adhesiveness to N-cadherin. Loss of PARP1 also increases the expression of other cell adhesion molecules in the embryonic brain, including *Nrcam*, *Dscaml1*, and *Astn1*. Interestingly, alterations in Reelin expression (Liu et al., 2001; Niu et al., 2008), cellular adhesion complex function (Seong et al., 2015), and neuronal density (Selemon and Goldman-Rakic, 1999) have all been previously associated with changes in dendritic arborization. Furthermore, prevention of CR cell programmed cell death results in increased dendritic complexity and spine density in pyramidal neurons, leading to altered excitatory/inhibitory balance in the brain (Riva et al., 2019). Additionally, our findings indicate that PARP1 loss alters the expression of genes that are important for regulating dendritic development and morphology. Together, these findings indicate a possible connection between a PARP1-mediated increase in CR cells with changes in neuronal density, which may underlie altered dendritic morphology. Further studies will be needed to explore the mechanistic links between these findings and PARP1 function.

Poly (ADP-ribose) polymerase 1 KO mice display endophenotypes associated with schizophrenia (Hong et al., 2019), and mutations in genes affecting PARylation have been linked to intellectual disability and episodic psychosis in humans (Najmabadi et al., 2011; Durmus et al., 2021). Likewise, defects in neurodevelopment and Reelin expression have been associated with schizophrenia and other neuropsychiatric disorders (Ayhan et al., 2011; Folsom and Fatemi, 2013; Muraki and Tanigaki, 2015). These observations raise the possibility that the brain development defects and schizophrenia-like behaviors in PARP1 KO mice may be a consequence of increased CR cell abundance. Intriguingly, both neuropsychiatric disorders (Selemon et al., 1998; Selemon and Goldman-Rakic, 1999; Chana et al., 2003) and reduced Reelin expression (Liu et al., 2001) have been associated with increased neuronal density in deeper layers of the cortex, similar to our findings in PARP1 KO mice. Moreover, increased mRNA expression of CSPGs has also been identified in schizophrenic patients (Pantazopoulos et al., 2010), suggesting increased expression of ECM genes due to PARP1 loss may be linked to increased neuronal density. Together, these findings suggest that the regulation of Reelin and ECM protein expression during embryogenesis is critical for normal development, and slight increases or decreases in Reelin levels or patterns of expression might impact brain development in ways that lead to neuropsychiatric disorders.

## Data Availability Statement

The datasets presented in this study can be found in online repositories. The names of the repository/repositories and accession number(s) can be found below: NCBI accessions: GSE171852, GSM5235796, GSM5235797, GSM5235798, GSM5235799, GSM5235800, GSM5235801, GSM5235802, and GSM5235803.

## Ethics Statement

The animal study was reviewed and approved by the University of Michigan Institutional Animal Care and Use Committee.

## Author Contributions

GC and MN designed the study, interpreted the data, and wrote the manuscript with the help of the other authors. MN performed most experiments and analyzed the data. JH performed the AFM experiments. MZ provided technical assistance. All authors contributed to the article and approved the submitted version.

## Conflict of Interest

GC is a scientific founder of Decibel Therapeutics, has an equity interest in and has received compensation for consulting. The company was not involved in this study. The remaining authors declare that the research was conducted in the absence of any commercial or financial relationships that could be construed as a potential conflict of interest.

## Publisher’s Note

All claims expressed in this article are solely those of the authors and do not necessarily represent those of their affiliated organizations, or those of the publisher, the editors and the reviewers. Any product that may be evaluated in this article, or claim that may be made by its manufacturer, is not guaranteed or endorsed by the publisher.

## Footnotes

1
www.usegalaxy.org

2
www.pantherdb.org
